# Case of Hernia Cerebri, Following a Compound Fracture of the Skull

**Published:** 1838-08-29

**Authors:** J. Forsyth Meigs

**Affiliations:** Resident Surgeon


### CLINICAL REPORTS.

### PENNSYLVANIA HOSPITAL.

*Case of Hernia Cerebri, following a compound Fracture of the Skull.*

[Reported by J. Forsyth Meigs, M. D., Resident Surgeon.]

Catharine K——, æt. 37, was admitted July 13th, 1838, for an injury of the head. It was stated, by the persons who conveyed her to the hospital, that a short time previous to entrance, she had slipped, while descending a flight of steps, with a bucket of water, and struck her head against the edge of one of them. On examination, a ragged wound, about three inches in length, was found on the right side near the junction of the temporal and parietal bones. The bone immediately beneath the wound was broken into two or three pieces, and driven in upon the brain. She was said to have lost much blood, but when I saw her, the bleeding was principally from beneath the depressed bone, and was not great. No symptoms of concussion or compression of the brain existed.

Pulse feeble; pupils natural; no paralysis. An attempt was made at once to raise the depressed portions of bone by means of the elevator, but these were so firmly jammed in as to make it impossible, until after the application of a small trephine. A perforation was made with this on the upper part of the exposed sound bone, after which the portions driven in upon the brain were readily removed. The dura mater was found to be slightly wounded, and a branch of the middle artery was divided. There was no effusion of blood between the dura mater and bone, and, on raising the latter, the hæmorrhage almost ceased. A strip of lint was placed over the divided vessel, and the wound was lightly covered. The pulse rose, and the patient, through an interpreter, stated her pain to be less after the removal of the bone. Perfect quiet, absolute diet, cold to head.

*July 14th.*—Is without fever or pain in the head; slept well; pulse moderate; pupils natural; states herself to be four months gone with child.

*July 16th.*—No pain in the head; pulse not excited; slight erysipelatous swelling of the scalp around the wound; simple dressings to wound; diet, and cold to head continued.

*July 17th.*—She was purged with calomel.

On the morning of the 19th, the erysipelatous appearance had disappeared, and she continued doing well till night, when she was observed to wander in her mind.

On the 20th, she complained of soreness of the wound, which, however, looked well and discharged healthy pus; skin pleasant; some heat of the head; pulse moderate, both in force and frequency; pupils natural; tongue clean; is correct in her replies to questions; simple dressing and cold to head continued. Towards evening she had a return of her delirium, and a twisting of the mouth and tongue to the left side was observed. Emplast. visicat. to back of neck; injection.

*July 21st.*—Delirium continues, but is not violent; rested badly; twisting of mouth and tongue continues, but has not much increased; cannot raise up her left arm from the bed or seize anything with her fingers; no paralysis of lower limbs. Calomel in small and repeated doses; minute doses of opium were afterwards combined with it, in order to prevent its operating upon the bowels.

*July 23d.*—She continued in the same state as on the 21st. The blister on the back of the neck not being very sore, a second was applied extending up upon the head.

*July 25th.*—Yesterday and to-day the patient has been much better. Twisting of the mouth is scarcely perceptible; free from delirium and pain; gums slightly sore; has partially regained the use of her arm, being able to raise it from the bed and to move slightly the fingers. Wound looks well and is closing rapidly; dressings of simple cerate continued, and nit. argent. applied to edges of the wound; calomel, and cold to the head omitted; bowels freely moved.

*August 1st.*—Since the last date the patient continued steadily improving. Yesterday a slight degree of puffiness or elevation at the wounded part was observed, which has to-day increased; pulsation of that part is also stronger; has to-day less power over the left arm than on 25th ult.; is exceedingly feeble; takes freely of mucilaginous articles of diet and milk; pressure just sufficient to give that degree of support to the part which it should naturally receive from the dura mater and bone, to be made with an adhesive strip.

*August 4th.*—The swelling and pulsation at wounded part continued the same; is free from delirium, though more dull; paralysis of left upper extremity has been again gradually increasing; perfect command over lower extremities; tongue clean; appetite good, and she complains of her debility; pulse very feeble; within the last two days has, at times, passed her urine involuntarily; soreness at wound but no pain in head. Bowels freely opened by injection; milk, gruels, and weak broth for food.

*August 9th.*—No change since the 4th except that the swelling on the side of the head has much increased since yesterday; pulsation stronger in it; is now about the size of a black walnut; no fever, and is correct in her answers; wound continues to be dressed with lint spread with cerate and the adhesive strip.

*August 10th.*—Swelling and pulsation not increased; a portion of the tumour, elliptical in shape, about an inch and a half long and three lines wide, is divested of the covering around the remaining part, and has the appearance of cerebral substance. No other alteration in the condition of the patient; same dressings and diet continued.

*August 12th.*—A piece of the inner table of the skull, of the size of the nail of the little finger, was removed from the posterior portion of the swelling. The swelling is somewhat softer; no alteration in the size of it, or in the strength of the pulsation; free discharge of pus from wound; the paralysis of the left upper extremity is perfect; the patient can move the left leg, but only partially; sensation, however, in the limb is normal; urine passed involuntarily; no fever, and answers correctly. Same diet and dressings continued; bowels opened with an injection.

*August 13th.*—Complains of some pain in the head; more tenseness in the tumour; no other alteration noticed. Same diet and dressings.

*August 14th.*—Complains of much pain in the head; is dull, and roused with difficulty; indisposed to reply to questions, though correct in her answers. Restless during the night, but no delirium; pulse slow and feeble; extremities cool; the tumour has considerably increased in size, in the direction of the ear; pulsation in it is strong. The same dressings continued; broth, gruels, &c., for diet; warmth to extremities; an aperient injection.

*August 15th.*—Complains of pain in the head; dull; indisposed to talk, but correct in answers; pulse fuller than yesterday; extremities warm; tumour about the size of a pullet's egg, though not of the same shape; pulsation very evident; the same diet and dressings continued.

*August 16th.*—Complains of much pain in the head; slightly delirious during the night; extremely dull; answers only in monosyllables, but correctly; complete paralysis of both the leg and arm of the left side; sensation not affected; pupils natural and sensible to light; pulse 80, and very feeble; appetite very good; tumour about the same size as yesterday; a portion of it about the size of a preserved black walnut, protrudes through the wound, and is covered by a vascular membrane supposed to be the pia mater, except in the elliptical space before mentioned, which has the appearance of the substance of the brain itself; adhesions exist between the investment of the tumour and the edges of the wound; and a narrow band of integument is stretched across the tumour, between the part covering the membrane, from that in which it is absent; the swelling continues beyond the boundaries of the original wound, elevating the integument, forming an irregular mass of the size before mentioned. The same dressings are continued to the tumour—broth, arrowroot and milk, for diet; bowels opened by an injection.

*August 17th.*—At 4 o'clock, A. M., stertorous breathing was noticed; patient could not be roused; complete paralysis of left side.

*August 18th.*—To-day can be somewhat more roused; tumour remains in same state.

*August 19th.*—Very feeble; pulse 158; respiration 44; skin hot over the whole surface; considerable discharge of pus from tumour, which at one spot presents the appearance of a commencing slough; can with great difficulty be roused so as to speak a few words very indistinctly; mouth very dry; wound dressed with adhesive strips and simple cerate; can swallow only a small quantity of barley water.

*August 20th.*—Gradually becoming more feeble; pulse over 180; respiration quick and laborious; skin still hot; can be roused sufficiently to protrude her tongue when desired; tumour increasing in size, with very considerable discharge of pus from a cavity situated between the integuments and bone; slough mentioned yesterday increasing; for nourishment takes merely a small quantity of arrow root and milk; involuntary discharge of urine; continue dressings as before.

*August 21st.*—Death at 3 o'clock, A. M. Autopsy at half-past 11 o'clock, A. M. Present, Drs. Pepper, Wallace and Smith. Tumour collapsed to one-fourth its previous size; considerable slough on the apex, the whole being of a dark brown colour. An incision was made across the top of the head, in the line of the coronal suture; another at right angles to this, touching the base of the tumour, and the integuments turned back so as to show clearly the wound of the scalp through which it protruded; this was an inch and a half in diameter, somewhat irregular in shape, but with its general outline circular, the edges very much thinned and of a dark slate colour; another cut was now made vertically through the tumour and the skull-cap subsequently removed; on attempting to strip off the dura mater from the brain it was found very much injected and strongly adherent, with an opening three-quarters of an inch in diameter, through which the hernia protruded, its edges being attached to the cut made by the trephine. It was now perfectly evident that the tumour consisted of the substance of the brain itself, arising by a narrow pedicle and expanding after passing through the bone. In removing the dura mater, a large abscess, occupying nearly the whole of the anterior and extending deeply into the middle lobe, was opened; this was lined by a false membrane the eighth of an inch thick, of a light brownish colour, and filled with thick yellow, inodorous pus; the brain immediately outside the membrane was softened, though at a little distance it appeared to be of the natural consistence. There was no effusion of blood either beneath the bone or under the dura mater; neither was there any apoplectic cavity as was generally supposed to be the case by Abernethy.

